# Using CT Data to Improve the Quantitative Analysis of ^18^F-FBB PET Neuroimages

**DOI:** 10.3389/fnagi.2018.00158

**Published:** 2018-06-07

**Authors:** Fermín Segovia, Raquel Sánchez-Vañó, Juan M. Górriz, Javier Ramírez, Pablo Sopena-Novales, Nathalie Testart Dardel, Antonio Rodríguez-Fernández, Manuel Gómez-Río

**Affiliations:** ^1^Department of Signal Theory, Networking and Communications, University of Granada, Granada, Spain; ^2^Department of Nuclear Medicine, “9 de Octubre” Hospital, Valencia, Spain; ^3^Clinical Medicine and Public Health Doctoral Program of the University of Granada, Granada, Spain; ^4^Biosanitary Investigation Institute of Granada, Granada, Spain; ^5^Department of Nuclear Medicine, “Virgen de las Nieves” University Hospital, Granada, Spain; ^6^Department of Nuclear Medicine, Lausanne University Hospital (CHUV), Lausanne, Switzerland

**Keywords:** quantitative analysis, multivariate analysis, florbetaben, Alzheimer's disease, support vector machine, positron emission tomography

## Abstract

^18^F-FBB PET is a neuroimaging modality that is been increasingly used to assess brain amyloid deposits in potential patients with Alzheimer's disease (AD). In this work, we analyze the usefulness of these data to distinguish between AD and non-AD patients. A dataset with ^18^F-FBB PET brain images from 94 subjects diagnosed with AD and other disorders was evaluated by means of multiple analyses based on *t*-test, ANOVA, Fisher Discriminant Analysis and Support Vector Machine (SVM) classification. In addition, we propose to calculate amyloid standardized uptake values (SUVs) using only gray-matter voxels, which can be estimated using Computed Tomography (CT) images. This approach allows assessing potential brain amyloid deposits along with the gray matter loss and takes advantage of the structural information provided by most of the scanners used for PET examination, which allow simultaneous PET and CT data acquisition. The results obtained in this work suggest that SUVs calculated according to the proposed method allow AD and non-AD subjects to be more accurately differentiated than using SUVs calculated with standard approaches.

## 1. Introduction

Alzheimer's disease (AD) is the most common neurodegenerative disease affecting more than 5 million people in the United States (Alzheimer's Association, 2018) and its prevalence in Europe was estimated at 5.05% (Niu et al., 2017). In addition, the number of AD patients is expected to increase during next decades because of the grow of the older population. Fortunately, the development of new drugs has greatly improved the patient's quality of life, especially when the disease is detected at an early stage. Thus, an early and accurate diagnosis of AD is crucial.

The diagnosis of AD is usually supported by neuroimaging data of different modalities. During the last decade, many research studies have demonstrated that both, structural and molecular imaging, can be successfully used to evaluate patients with AD, including early stages and prodromal AD (Johnson et al., 2012). Structural neuroimaging data such as magnetic resonance imaging (MRI) or computed tomography (CT) allow us to estimate the global cerebral volume, which was found to significantly correlate with the rate of change in mini-mental state examination scores, evidencing clinical relevance to this marker in the disease progression (Frisoni et al., 2010; Khedher et al., 2015). In addition, MRI and CT data can be used to exclude treatable or reversible causes of dementia (normal-pressure hydrocephalus, subdural hematoma, tumors, etc.).

On the other hand, molecular neuroimages have been widely used in differential diagnosis of dementia. For example, Single Photon Emission Computer Tomography (SPECT) or Positron Emission Tomography (PET) have been demonstrated as valuable tools not only to separate AD patients and controls (Segovia et al., 2010; Rathore et al., 2017) but also to monitor the progression of AD (Hanyu et al., 2010). Probably, the most common molecular neuroimaging modality for AD diagnosis is the well-known ^18^F-Fludeoxyglucose (FDG) PET. These images allow us to analyze the glucose brain metabolism and that way to estimate the neurodegeneration of certain regions of the brain (Illán et al., 2011; Perani et al., 2014; Cabral et al., 2015).

Conversely to ^18^F-FDG PET, amyloid imaging focuses on the amyloid beta deposits that characterize AD. During last years, several radiotracers have been proposed to examine these AD hallmarks. The N-methyl-[^11^C]2-(4′-methylaminophenyl)-6-hydroxybenzothiazole, more commonly referred to as Pittsburgh Compound B (PIB), is an amyloid focused radiotracer traditionally used for this purpose (Klunk et al., 2004). This drug is a radioactive analog of thioflavin T, which binds to amyloid plaques with high affinity, however, its reduced half-life (only 20 min) greatly limits its application (Klunk and Mathis, 2008). Recently, new ^18^F-labeled tracers with similar efficacy to PIB and longer half-life have been FDA approved: ^18^F-florbetapir in 2012, ^18^F-flutemetamol in 2013 and ^18^F-florbetaben (FBB) in 2014. The validity of these radiotracers is supported by recent studies (Landau et al., 2014; Rice and Bisdas, 2017) that emphasize the added value of these radiotracers in discriminating between AD and non-AD patients (Ceccaldi et al., 2018).

In this work, we analyze ^18^F-FBB PET data from AD and non-AD patients using univariate and multivariate techniques. In order to improve the diagnosis of AD we propose to include in the analysis the information about gray matter neurodegeneration provided by CT images. This approach takes advantage of the majority of PET images are acquired on scanners that allow simultaneous PET and CT data acquisition. Specifically, we propose to calculate standardized uptake values from ^18^F-FBB PET data using only voxels belonging to gray matter in CT images. Previous works have followed similar approaches (Villemagne et al., 2015; Rullmann et al., 2016) but in those cases non-gray-matter voxels were discarded only for the reference region and they were determined by means of MRI images. The proposed approach was evaluated using a dataset with ^18^F-FBB PET and CT scans from 94 subjects acquired during a longitudinal study carried out in two hospitals from the Spanish National Health System. The results suggest that using CT data along with ^18^F-FBB PET neuroimages improves up to 7% the accuracy of separating AD and non-AD patients, compared with using only PET data.

## 2. Materials and methods

### 2.1. Participants

Ninety-four (94) subjects with cognitive impairments were recruited in the Cognitive Behavioral Unit of two different tertiary hospitals: the Virgen de las Nieves hospital in Granada, Spain (72 patients) and the 9 de Octubre hospital in Valencia, Spain (22 patients).

Patients were recruited according to the following clinical criteria: patients with persistent or progressive unexplained MCI Albert et al. (2011); Johnson et al. (2013), defined according to revised Petersen criteria (Winblad et al., 2004); patients fulfilling core clinical criteria for possible AD but an atypical clinical course with no documented progression in the patient's records; patients fulfilling these core clinical criteria but with cerebrovascular comorbidity, concomitant pharmacologic, neurologic, or cognitive components (mixed etiology); and those with a history of progressive dementia and atypically early age at onset (≤ 65 years). All patients fulfilled clinical appropriate use criteria for ^18^F-FBB PET scan according to international consensus (Johnson et al., 2013). Exclusion criteria were: the presence of a metabolic disorder (hypothyroidism, vitamin B12 or folic acid deficiencies), psychiatric pathology (schizophrenia or depression), MRI-diagnosed cerebrovascular disease (infarction or hemorrhage), neurologic disease affecting gnosis (Parkinsonian syndrome, epilepsy, etc.), pregnancy, glycemia > 160 mg/dL, history of substance abuse, or age < 18 years.

Patients were evaluated using standardized neuropsychological examinations that assessed the orientation, attention, memory, executive function, language, visual and constructive functions and behavior (Carnero Pardo, 2007). In addition, a ^18^F-FBB PET and a CT scan were acquired for each patient. The imaging protocol in both centers complied with international guidelines (Minoshima et al., 2016). Specific details are given in Table 1.

**Table 1:** Protocol details to acquire neuroimaging data.

	**Center A**	**Center B**
Camera	GE Discovery STE	Siemens Biograph 16
Patient position	Resting, with closed eyes	Resting, with closed eyes
Operation	3D mode	3D mode
Filtering	Z-Axis standard	Z-Axis standard
Dose	300 MBq	300 MBq
Acq. start	90 min post injection	90 min post injection
Acq. duration	20 min	20 min
Matrix size (FBB)	168 × 168	168 × 168
Slice thichness (FBB)	4.01 mm	4.06 mm
Number of slices	70	70
Voxel size	16.08 (mm^3^)	16.48 (mm^3^)
Reconstruction	VUE Point (5 it, 35 sub)	Gaussian + OS-OM (6 it, 21 sub)
CT Parameters	Low-dose, 80 mAs, 120 kV	Low-dose, 50 mAs, 120 kV
Matrix size (CT)	512 × 512	512 × 512
Slice thichness (CT)	1 mm	1 mm
Corrections	Scatter; CT attenuation; well counter sensitivity and activity; delayed event subtraction and normalization	Scatter; CT attenuation; slice coincidence location with CT

After at least 1 year of follow-up, experienced neurologists established a final diagnosis for each patient on the basis of neuropsychological examinations, the visual assessment of the neuroimaging data and the clinical evolution of the patient. Two subgroups were defined: (i) AD patients and (ii) healthy subjects or patients with diseases other than AD. Table 2 shows the group distribution and some demographic details of the patients. Note that the second group is very heterogeneous and includes patients with Parkinson's disease, progressive supranuclear palsy and psychiatry disorders among other conditions.

**Table 2:** Demographic details of the patients considered in this work (μ and σ stand for the average and the standard deviation respectively).

		**Sex**		**Age**		
	**#**	**M**	**F**	**μ**	**σ**	**range**
AD	51	23	28	63.43	6.32	49–74
Non-AD	43	28	15	62.91	8.27	42–79

Each patient (or a close relative) gave written informed consent to participate in the study and the protocol was accepted by the Ethics Committee of the “Virgen de las Nieves” hospital (Granada, Spain) and the “9 de Octubre” hospital (Valencia, Spain). All the data were anonymized by the clinicians who acquired them before being considered in this work.

### 2.2. Data preprocessing

CT brain images were segmented using the unified segmentation algorithm (Ashburner and Friston, 2005) implemented in Statistical Parametric Mapping (SPM) version 12. This algorithm allows the separation of gray matter, white matter and cerebrospinal fluid tissues from CT images. The ^18^F-FBB PET images were also registered to a common space using SPM. This procedure made use of the deformation fields obtained during the segmentation of the CT data in order to achieve a more accurate transformation (Ashburner and Friston, 2007). As a result, we got brain images in Montreal Neuroimaging Institute (MNI) space with 121 × 141 × 121 voxels of 1.5 × 1.5 × 1.5 mm each.

### 2.3. Regions of interest

**Figure 1:**
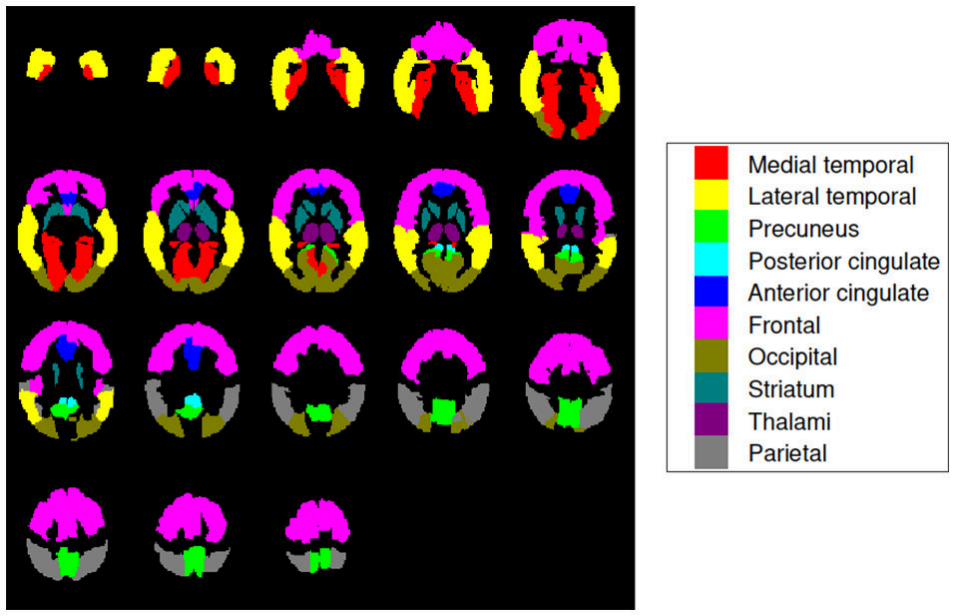
Brain map encoding with colors the regions of interest used in this work. They are known to be particularly useful for AD diagnosis and are widely used in literature (Rodriguez-Vieitez et al., 2016). Axial slices at −36, −30, −24, …, 66 mm from the anterior commissure are shown.

Ten (10) regions of interest (ROIs) were defined to analyze our ^18^F-FBB PET data: medial temporal, lateral temporal, precuneus, posterior cingulate, anterior cingulate, frontal, occipital, striatum, thalami, and parietal (Rodriguez-Vieitez et al., 2016). Locations and sizes can be seen in Figure 1. These regions are frequently associated to AD in the literature and allow comparing our results with the ones obtained by other works (Villemagne et al., 2012; Daerr et al., 2016; Tiepolt et al., 2016; Tuszynski et al., 2016; Bullich et al., 2017). In order to parcel these target regions in our brain images the Automatic Anatomical Labeling (AAL) atlas was used (Tzourio-Mazoyer et al., 2002).

### 2.4. Quatification of ^18^F-FBB PET data using structural information

In the clinical practice, neuroimaging data are usually analyzed in terms of standardized uptake values (SUV), which are often given as a ratio of the uptake of a reference region (SUVR). Different regions have been propose to be used as reference to calculate SUVRs from amyloid PET data (Brendel et al., 2015; Klein et al., 2015a,b; Kimura et al., 2016; Shokouhi et al., 2016). Despite there is no general consensus, the use of the whole cerebellum (Daerr et al., 2016; Bullich et al., 2017) or the cerebellar gray matter (Villemagne et al., 2015) is usually accepted. The SUVR for a given region, *k*, could be computed as:

(1) $$\begin{array}{l} SUVR_{k}=\frac{N_{r}\sum_{i=1}^{N_{k}}x_{i}}{N_{k}\sum_{j=1}^{N_{r}}x_{j}} \end{array}$$

where *x*_*i*_ is the intensity of the *i*-th voxel belonging to region *k*, with *i* ∈ [1, 2…*N*_*k*_] and similarly, *x*_*j*_ stands for the intensity of the *j*-th voxel belonging to a reference region, with *j* ∈ [1, 2…*N*_*r*_]. In this work, we used the whole cerebellum as reference region thus, SUVRs for a given subject were weighted by the mean cerebellar intensity of that subject. This analysis is somewhat similar to the visual examination of the data traditionally performed by experienced clinicians.

Instead of SUVR described by Equation 1, we propose to use a similar measure that also takes into account structural data. Specifically we propose to compute SUVRs using only voxels belonging to gray matter, i.e., those whose position corresponds to gray-matter voxels in CT data. That way we consider not only the amyloid deposits but also the brain injury caused by the disorder.

### 2.5. Fisher's discriminant analysis

The Fisher's discriminant ratio, *J*, (Theodoridis and Koutroumbas, 2008) is a statistical measure widely used to maximize the differences of means in between two or more classes respective to the within class variance (Lopez et al., 2009). Mathematically it is defined as:

(2) $$\begin{array}{l} J\left(\text{w}\right)=\frac{{\text{w}}^{T}S_{B}\text{w}}{{\text{w}}^{T}S_{W}\text{w}} \end{array}$$

where **w** represent a direction in the data space and *S*_*B*_ and *S*_*W*_ are respectively the “between classes” and the “within classes” scatter matrices. Note that scatter matrices are proportional to covariance matrices and, when only 2 classes are considered, *S*_*B*_ can be expressed as:

(3) $$\begin{array}{l} S_{B}=\left(\mu_{1}-\mu_{2}\right){\left(\mu_{1}-\mu_{2}\right)}^{T} \end{array}$$

where μ_*i*_ denotes the mean of the samples belonging to the *i*-th class. This analysis was not applied to individual voxels (each possible direction in the image space would correspond to a specific voxel position) but to the SUVRs of the ROIs defined in section 2.3. Thus, *J* was computed as:

(4) $$\begin{array}{l} J_{r}=\frac{{\left(\mu_{1}^{\left(r\right)}-\mu_{2}^{\left(r\right)}\right)}^{2}}{{\left(\sigma_{1}^{\left(r\right)}\right)}^{2}+{\left(\sigma_{2}^{\left(r\right)}\right)}^{2}} \end{array}$$

where $\mu_{i}^{\left(r\right)}$ and $\sigma_{i}^{\left(r\right)}$ are, respectively, the mean and the standard deviation of the SUVR of region *r* for subjects belonging to the *i*-th class.

### 2.6. Support vector machine

A binary classification method is a statistical procedure intended to assign a binary label (defining a category or group) to unseen patterns represented by a set of features. To this end, supervised methods build a function *f*:ℝ^*D*^ → ±1 using a set of known patterns, **x**_*i*_ and their labels, *y*_*i*_ (training data):

(5) $$\begin{array}{l} \left({\text{x}}_{1},y_{1}\right),\left({\text{x}}_{2},y_{2}\right),\ldots ,\left({\text{x}}_{N},y_{N}\right)\in \left({\mathbb{R}}^{k}\times \left\{\pm 1\right\}\right) \end{array}$$

Support Vector Machine (SVM) is a supervised classifier derived from the statistical learning theory (Vapnik, 1998). In SVM the classification function is built using a hyperplane, called maximal margin hyperplane, that has the largest distance to the closest training data pattern of any group:

(6) $$\begin{array}{l} g\left(\text{x}\right)={\text{w}}^{T}\text{x}+w_{0}=0, \end{array}$$

where **w** is the weight vector, orthogonal to the decision hyperplane, and *w*_0_ is the threshold. SVM is able to work in combination with kernel approaches when the linear separation of the data is not possible (Müller et al., 2001). Once the hyperplane is computed the classifier assigns a group label to each new pattern according to the side of the hyperplane where it is.

In our experiments the cost parameter, *C*, was fixed to the commonly accepted value of *C* = 1 and only linear kernels were used. The evaluation of the classification performance was carried out using a 10-fold cross-validation scheme (Varma and Simon, 2006). Given that we have data from 94 subjects, each fold uses 85 samples for training and 9 for test. In the training step of each fold, SUVRs or voxel intensities from each training subject and a binary label determining the group the subject belongs to (AD or non-AD) were used as input data (variables **x**_*i*_ and *y*_*i*_ in Equation 5). In the test step, the classifier was used to estimate a label for each test subject (represented by its SUVRs or voxel intensities). The estimated labels were then compared with the real ones to assess the classification performance.

### 2.7. ROC analysis

In a classification procedure, the trade off between sensitivity and specificity can be analyzed through a Receiver Operating Characteristic (ROC) curve (Brown and Davis, 2006). In these plots, each point represents a sensitivity/specificity pair corresponding to a particular decision threshold. The upper left corner correspond to a sensitivity and specificity of 100%, therefore, the closer the ROC curve is to the upper left corner, the highest accuracy. The area under the curve (AUC) allows measuring how close is the solution to the optimal one and is frequently used as a measure of the classification performance.

## 3. Experiment and results

**Figure 2:**
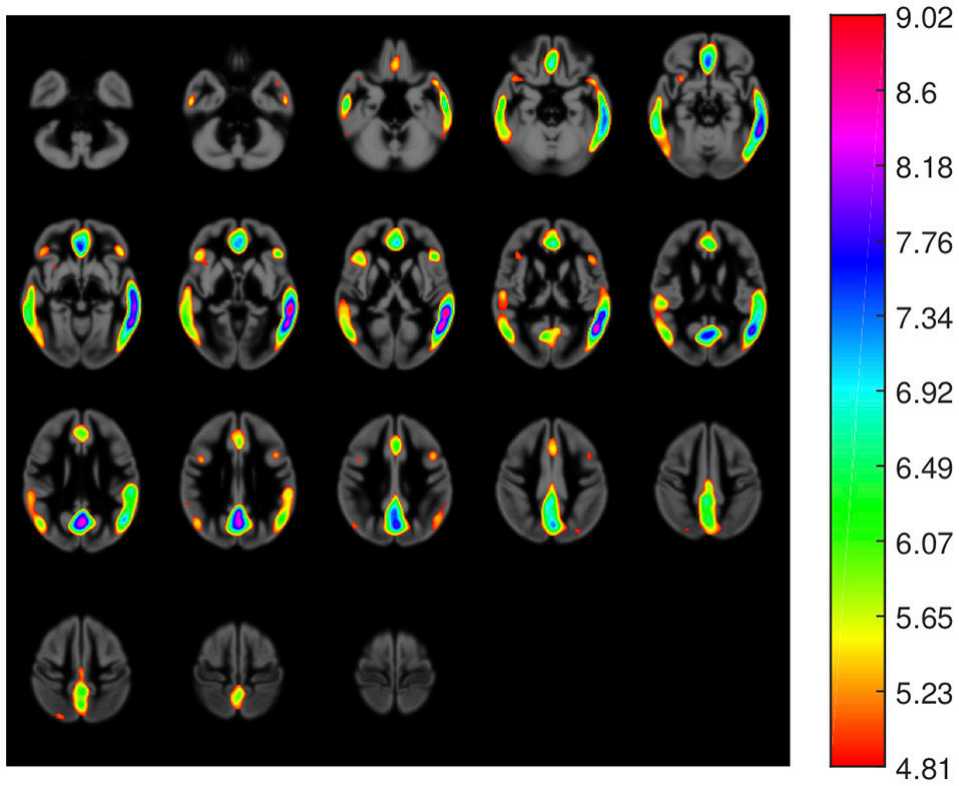
Areas with significant differences (*p* < 0.05, FWE) between groups in ^18^F-FBB PET data. The color scale codifies the *t*-statistic values (values below 4.81 are not significant).

First, we carried out a *t*-test analysis on SPM to look for group differences between AD and non-AD subjects for both, ^18^F-FBB PET and CT data. As sugested in Friston et al. (2006), a smoothed version of the brain images (Gaussian filter of 8 mm FWHM) was used. Results for PET images are shown in Figure 2. In this case, we evaluated the hypothesis that data from AD patients have higher intensity than those from non-AD subjects (AD patients are expected to have a greater amyloid-beta concentration). Voxels with significant differences (*p* < 0.05, FWE) between both groups are shown in a specific color which depends on its *t*-statistic. In order to determine if the colored regions match with target regions described in section 2.3, we calculated the percentage of those regions covered by colored voxels. The results are shown in Table 3. No significant effects were found for CT data. In this case, only the gray-matter was used and two hypotheses were evaluated: non-AD group has lower intensity than AD group (same as for PET images) and AD group have lower intensity than non-AD group. The latter hypothesis was the most plausible for CT images since one might expect a greater neurodegeneration in AD patients.

**Table 3:** AD target regions and areas with significant differences between AD and non-AD patients in ^18^F-FBB PET data.

**Region name**	**Region size**	**Area with significant diff**.
Medial temporal	47171 mm	432 mm (0.92 %)
Lateral temporal	94907 mm	36732 mm (38.70 %)
Precuneus	24338 mm	9693 mm (39.83 %)
Posterior cingulate	2813 mm	551 mm (19.57 %)
Anterior cingulate	9603 mm	5346 mm (55.67 %)
Frontal	193730 mm	10424 mm (5.38 %)
Occipital	50117 mm	6248 mm (12.47 %)
Striatum	16326 mm	0 mm (0.00 %)
Thalami	7422 mm	0 mm (0.00 %)
Parietal	50763 mm	4715 mm (9.29 %)

*The differences were determined by means of a t-test analysis*.

**Figure 3:**
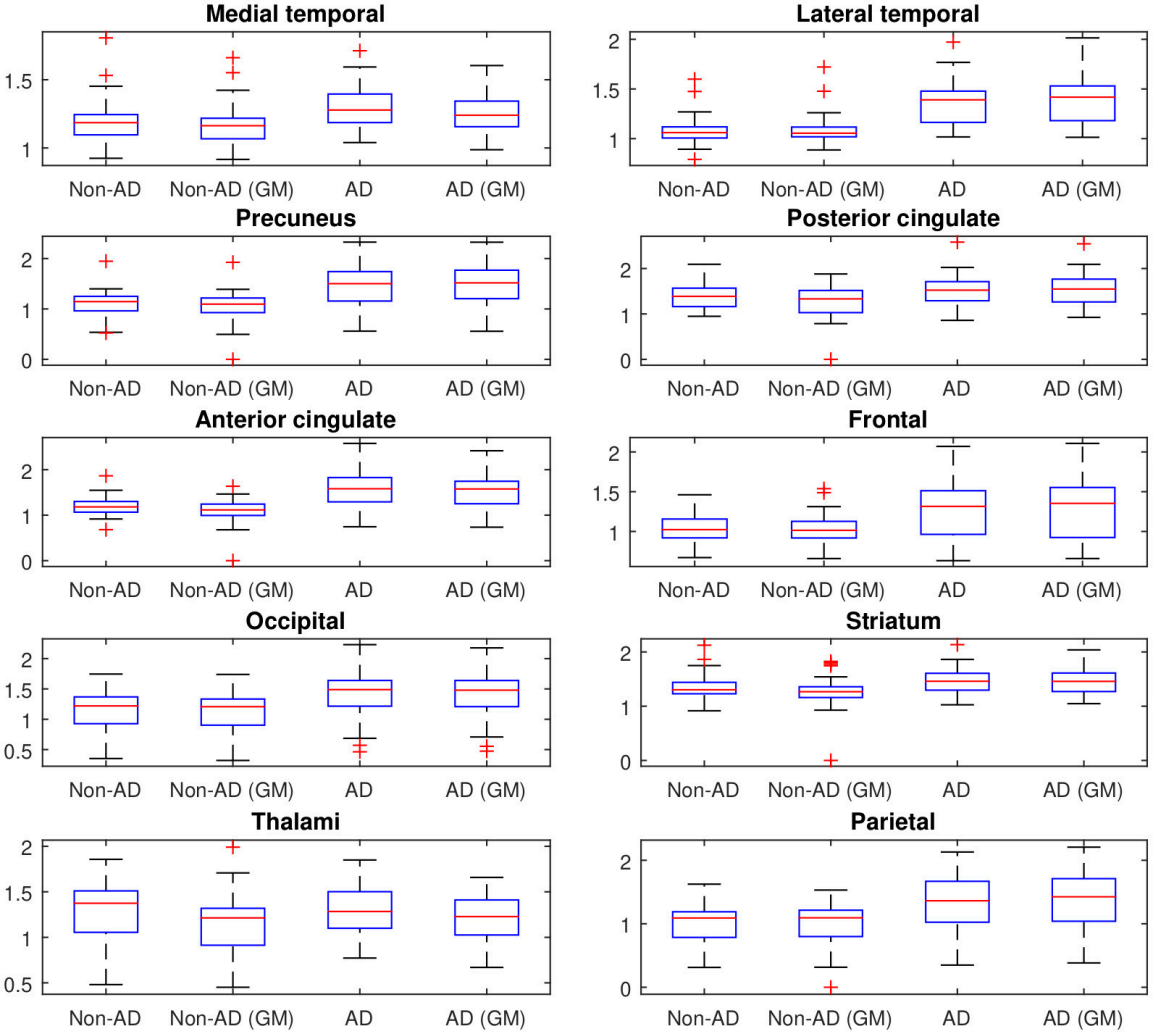
SUVR of the 10 target regions described in section 2.3. The values are grouped into 4 groups according to: (i) the class they belong to (AD or non-AD) and (ii) how they were calculated (using all voxels (classical approach) or using only gray-matter voxels (proposed approach)). On each blue box, the central mark indicates the median, and the bottom and top edges of the box indicate the 25th and 75th percentiles, respectively.

Afterwards, the advantages of computing SUVRs from ^18^F-FBB PET data using only the gray-matter voxels were evaluated. Figure 3 shows the median SUVR of each target region grouped into four groups according to: i) the class they belong to (AD or non-AD) and ii) how they were calculated: using all voxels (classical approach) or using only gray-matter voxels (proposed approach). The F-statistic (ANOVA) and corresponding *p*-value were computed to determine whether AD and non-AD subjects have different mean on target regions. Results using the classical and the proposed procedure to calculate SUVRs are given in Table 4.

**Table 4:** F-statistics and corresponding *p*-values to determine whether AD and non-AD patients have different mean on target regions.

	**Classical SUVR**		**Proposed SUVR**	
**Region**	**F-statistic**	***p*-value**	**F-statistic**	***p*-value**
Medial temporal	7.9922	0.0058	6.6137	0.0117
Lateral temporal	50.3387	0.0000	53.6227	0.0000
Precuneus	27.7957	0.0000	34.3257	0.0000
Posterior cingulate	4.5438	0.0357	13.6022	0.0004
Anterior cingulate	37.1421	0.0000	41.8245	0.0000
Frontal	15.2235	0.0002	18.1024	0.0001
Occipital	17.4945	0.0001	17.9269	0.0001
Striatum	6.2384	0.0143	12.1945	0.0007
Thalami	0.0437	0.8349	0.7259	0.3964
Parietal	19.0551	0.0000	22.6125	0.0000

**Figure 4:**
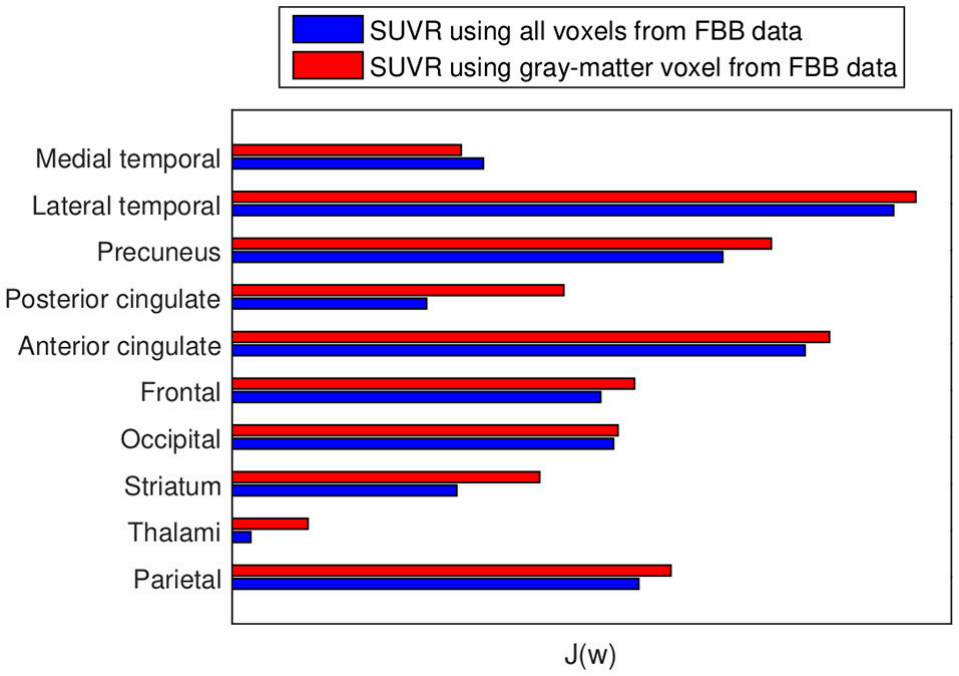
Fisher's discriminant ratio for SUVRs of target regions. Blue: Rates for SUVRs computed using all the voxels in the region. Red: Rates for SUVRs calculated using only gray-matter voxels. Larger values mean larger distances between groups.

The advantages of proposed SUVRs were also assessed by means of the Fisher's discriminant analysis. J_*r*_ values (Equation 4) were computed to rate the usefulness of SUVRs of target regions when separating AD and non-AD subjects. Figure 4 allows us to compare the J_*r*_ values computed using all brain voxels with those that considered only gray-matter voxels.

**Figure 5:**
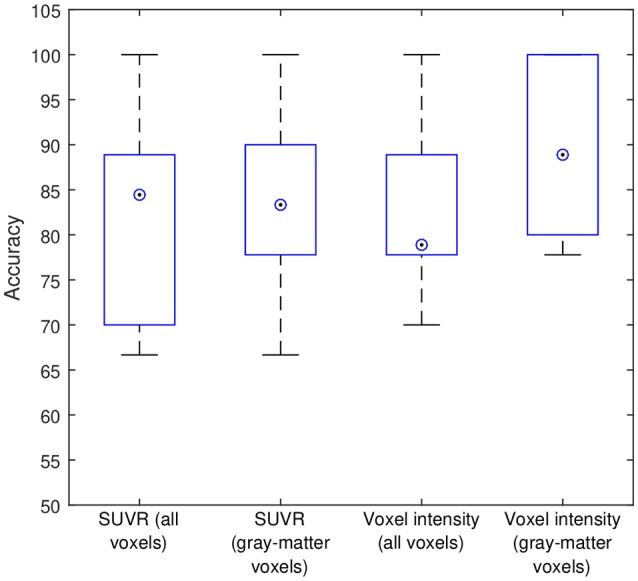
Intermediate accuracies obtained in the cross-validation procedure. Blue boxes and circled dots represent accuracies' range and median respectively.

**Figure 6:**
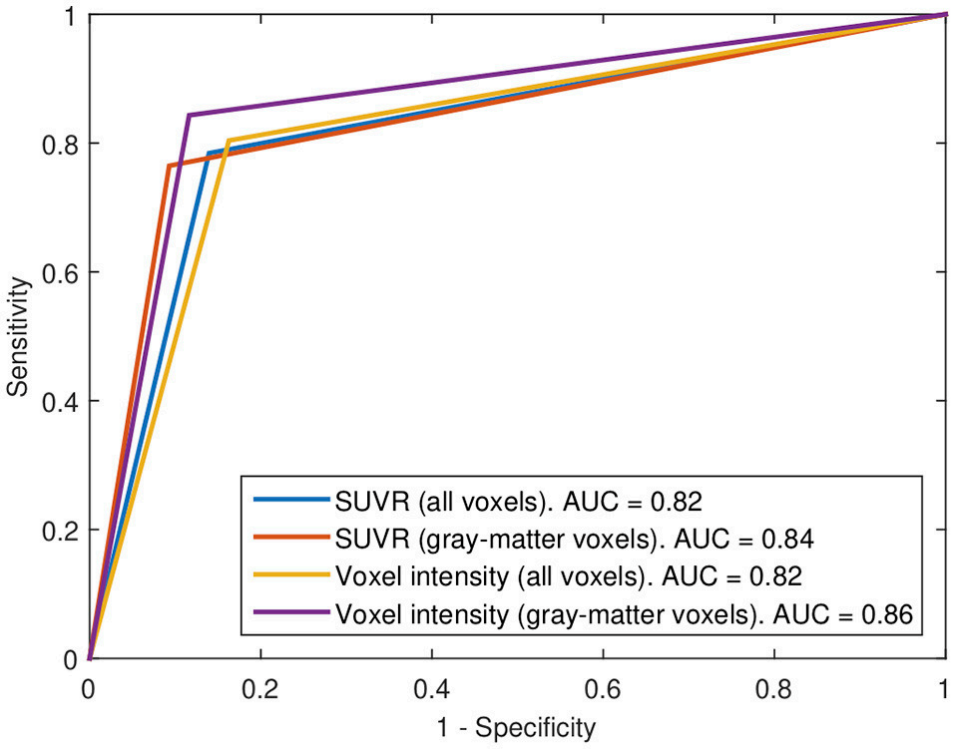
ROC curves for the 4 SVM procedures implemented in this work.

Subsequently, our data were analyzed in terms of their usefulness to separate AD and non-AD patients using SVM classification. Specifically, we estimated the accuracy, sensitivity and specificity of a SVM classifier that separates the groups using ^18^F-FBB PET data. Two approaches were applied: (i) using SUVR of target regions as feature and (ii) using the intensity of all the voxels in brain images as feature. In both cases we compared the classification results when using or not the CT data to exclude non-gray-matter voxels. For the approach using all the voxels in brain images, the intensity of the voxels was referenced to the mean uptake of the whole cerebellum. This is similar to the intensity normalization performed during the calculation of SUVRs but, in this case, the normalization was individually applied to each voxel. The classification results are shown in Table 5 and Figure 5. The trade off between sensitivity and specificity of the SVM analyses was examined by means of ROC curves. They are shown along with the AUC in Figure 6.

**Table 5:** Classification measures obtained by a SVM classifier when separating AD and non-AD subjects using ^18^F-FBB PET data.

**Data used as feature**	**Accuracy (%)**	**Sensitivity (%)**	**Specificity (%)**
SUVR (all voxels)	81.91	78.43	86.05
SUVR (gray-matter voxels)	82.98	76.47	90.70
Voxel intensity (all voxels)	81.91	80.39	83.72
Voxel intensity (gray-matter voxels)	86.17	84.31	88.37

**Figure 7:**
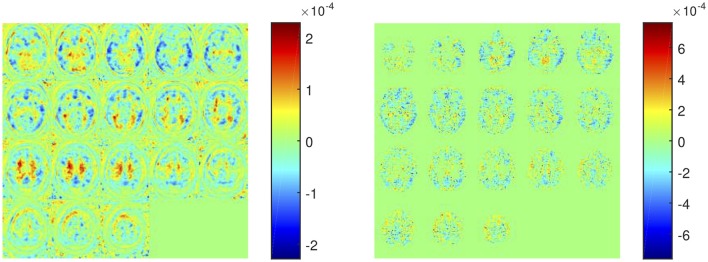
Weight assigned to each voxel by a SVM classifier trained using the intensity of all voxels as feature **(left)** and using the intensity of gray-matter voxels as feature **(right)**.

The weight map calculated by SVM (parameter **w** in Equation 6) allows us to examine the importance of each feature in the classification procedure. SVM weights from systems using SUVRs as feature are shown in Table 6 whereas those calculated by systems using voxel intensities as feature are shown in Figure 7. Note that in former systems only 10 regions were used, thus only 10 weights were calculated. Similarly, the systems using voxel intensities as feature computed as many weights as voxels were used.

**Table 6:** Weight assigned to each target region by a SVM classifier that used the SUVRs of those regions as feature.

**ROI**	**All voxels**	**Gray-matter voxels**
Medial temporal	0.09	0.17
Lateral temporal	−1.90	−2.28
Precuneus	−0.68	−0.88
Posterior cingulate	0.37	0.50
Anterior cingulate	−0.81	−0.90
Frontal	1.10	1.45
Occipital	0.54	0.55
Striatum	0.31	0.48
Thalami	0.29	−0.16
Parietal	0.12	0.16

*Two approaches to calculate SUVRs were assessed: using all the voxels in the region (center column) and using only gray-matter voxels in the region (right column)*.

All the experiments were carried out on Matlab using its statistical toolbox and specific *ad hoc* routines.

## 4. Discussion

The experiments carried out in this work corroborated that ^18^F-FBB PET is an useful neuroimaging modality to assist the diagnosis of AD. Both, univariate and multivariate analyses indicated that these data allow us to separate AD and non-AD subjects with high accuracy. In addition, the regions commonly focused on AD diagnosis show large group differences in ^18^F-FBB PET neuroimages. According to the results shown in Table 3, lateral temporal, precuneus, posterior and anterior cingulate have significant differences between groups. Additionally, the former region is the more important one to separate the AD and non-AD subjects as suggested by the results shown in Table 6. The results shown in these tables should be carefully interpreted. Table 3 contains the percentage of each ROI with significant differences (*p* < 0.05, FWE) whereas Table 6 shows the weights assigned by a SVM classifier to those regions when the SUVRs of those regions were used to train the classifier. Thus, frontal is an important region in the separation problem because the classifier assigned it a high weight (relatively high compared with other weights). However, only about 5.38% of voxels in this region (according to the AAL atlas) showed significant differences in the *t*-test. This suggest that the importance of frontal in the separation problem is not homogeneous throughout the region and some frontal “subregions” are more important than others. It should be noted that frontal was defined as a big region (with a volume of almost 200 cm^3^ in the AAL atlas), more than 10 times larger than precuneus, a small region with high significance but with not such a high weight. Lateral temporal and anterior cingulate are also two important regions because of their large absolute value in Table 6. SVM weights concern the side of the hyperplane where patterns are placed. Thus, negative weights are associated to regions that characterize non-AD subjects (they “move” patterns toward the non-AD space) whereas positive weights are associated to AD subjects (they “move” patterns toward the AD space) (Caragea et al., 2001). Observe that using only gray-matter voxels made the weights more positive or more negative for all regions except for thalami. This suggests that ^18^F-FBB PET data contain no important information to separate the groups in this region. This is consistent with *t*-test results, which found no significant differences in thalami.

The *t*-test analysis has drawn two clear conclusions: (i) there are significant group differences in ^18^F-FBB PET neuroimages and, (ii) there are no significant group differences in CT data. The latter conclusion can be explained by the composition of the non-AD group, which contains a large proportion of subjects with other diseases, including parkinsonian disorders, that could have structural changes similar to AD. The lack of significant group differences in CT data may also be due to the neuroimaging modality itself (Gado et al., 1983). Although a number of studies (Grundman et al., 2002; Rathore et al., 2017) have reported volumetric differences between AD and non-AD patients in MRI data, the use of CT neuroimages to this purpose have been poorly studied.

In this work we propose to use SUVRs from ^18^F-FBB PET neuroimages that also considerer the gray matter neurodegeneration in order to improve the diagnosis of AD. In most cases, this information can be extracted from CT data in a inexpensive and efficient way, since most of the scanners used for PET are combined PET/CT devices. Specifically, we propose to discard those voxels from ^18^F-FBB PET images not belonging to gray matter in CT images and therefore, calculate SUVRs using only the gray-matter voxels. The idea of discarding non-gray-matter voxels was used in previous works (Villemagne et al., 2015; Rullmann et al., 2016) to calculate the SUV of the reference region or to perform intermediate corrections. Here, we propose to apply it to the SUV calculation of all the regions and, to this end, we propose to use CT data due to its greater availability. This way to compute SUVs is similar to the one used in Gonzalez-Escamilla et al. (2017) but we used CT instead MRI images. The results obtained in this work suggest that the proposed approach allows separating AD and non-AD patients more accurately than using standard methods for SUVR calculation. As shown in Figure 4, for 9 out of 10 ROIs the computation of the SUVR that considered only the gray matter separated the patient groups more than the SUVR computed using standard methods. These results were corroborated by ANOVA and SVM analyses. Tables 4, 5 show that mean differences between groups are greater (higher F-statistics and lower *p*-values) and accuracy rates in SVM classification are larger when SUVRs were computed using only gray-matter voxels.

SVM classification performed an accurate separation (accuracy above 80% for the 4 studied feature sets) of the groups, which is particularly important if we take into account that the separation of AD patients from other neurological disorders is more difficult than distinguishing between AD patients and healthy subjects (as mentioned before non-AD group contains a large number of patients with other disorders). Although the heterogeneity of non-AD group could be seen as a limitation of our study, this approach is, in our opinion, more interesting because it is very similar to the clinical problem where clinicians usually take care of non-healthy subjects and should differentiate between AD and other disorders. The obtained accuracy rates suggest that ^18^F-FBB PET data contain useful biomarkers to develop computer-aided diagnosis systems for AD. Anyway, the analysis of the reported accuracy rates should consider potential labeling errors inherent in all diagnostics.

The proposed approach to calculate SUVRs must not be confused with Partial Volume Effect correction (PVEc) methods (Erlandsson et al., 2012; Matsubara et al., 2016; Rullmann et al., 2016). In fact, the application of those corrections are compatible with the way to calculate SUVRs that we are proposing. In this work, we decided not using PVEc methods due to: (i) presently, they are not routinely applied, neither in clinical nor in research settings and (ii) these techniques depend on a range of model assumptions and may result on noise amplification (Erlandsson et al., 2012; Greve et al., 2016; Gonzalez-Escamilla et al., 2017).

## 5. Conclusions

In this work we have proposed to compute SUVRs from amyloid-PET imaging considering also structural data. Specifically, we proposed to use only gray-matter voxels, estimated through CT images, to calculate SUVRs. In order to evaluate the proposed approach, different experiments based on *t*-test, ANOVA, FDR and SVM were carried out. A dataset with ^18^F-FBB PET and CT brain images from 94 subjects diagnosed with AD and other disorders was used for evaluation purposes. The results of those experiments suggest that the proposed method to calculate SUVRs allows separating AD and non-AD subjects more accurately than SUVRs calculated by standard methods. Additionally, the results obtained in this work corroborated that ^18^F-FBB PET data are good biomarkers to estimated brain amyloid deposits and are useful to diagnose AD.

## Author contributions

RS-V, PS-N, NT-D, AR-F, and MG-R: Data collection; FS, RS-V, JG, JR, PS-N, and MG-R: Conception and design of the work; FS, JG, and JR: Data analysis; FS, RS-V, JG, JR, PS-N, and MG-R: Result interpretation; FS: Drafting the article; FS, RS-V, JG, JR, PS-N, and MG-R: Critical revision of the article.

### Conflict of interest statement

The authors declare that the research was conducted in the absence of any commercial or financial relationships that could be construed as a potential conflict of interest.
